# First-in-human study of an optimized, potential kit-type, SSTR antagonist ^68^Ga-DATA^5m^-LM4 in patients with metastatic neuroendocrine tumors

**DOI:** 10.7150/thno.94521

**Published:** 2025-01-20

**Authors:** Jingjing Zhang, Lukas Greifenstein, Vivianne Jakobsson, Elcin Zan, Andre Klega, Frank Rösch, Christian Landvogt, Corinna Mueller, Richard P. Baum

**Affiliations:** 1 Department of Diagnostic Radiology, Yong Loo Lin School of Medicine, National University of Singapore, Singapore, Singapore.; 2 Theranostics Center of Excellence, Yong Loo Lin School of Medicine, National University of Singapore, 11 Biopolis Way, Helios, Singapore 138667, Singapore.; 3 Clinical Imaging Research Centre, Centre for Translational Medicine, Yong Loo Lin School of Medicine, National University of Singapore, Singapore, Singapore.; 4 Nanomedicine Translational Research Program, NUS Center for Nanomedicine, Yong Loo Lin School of Medicine, National University of Singapore, Singapore, Singapore.; 5 Curanosticum Wiesbaden-Frankfurt, Center for Advanced Radiomolecular Precision Oncology, Wiesbaden, Germany.; 6 Academy for Precision Oncology, International Centers for Precision Oncology (ICPO), Wiesbaden, Germany.; 7 Department of Radiology, New York University Langone Medical Center, New York, NY, USA.; 8 Department of Chemistry, TRIGA, Johannes Gutenberg University, Mainz, Germany.

**Keywords:** SSTR antagonist, ^68^Ga-DATA^5m^-LM4, first-in-human, neuroendocrine tumors (NETs), somatostatin receptor, PET/CT

## Abstract

Radiolabeled somatostatin receptor (SSTR) agonists ^68^Ga-DOTA-TATE and ^68^Ga-DOTA-TOC are widely applied for imaging of patients with neuroendocrine tumors (NETs). Preclinical and preliminary clinical evidence has indicated that SSTR antagonists perform better for NET imaging. In this study, we assessed the feasibility of using a new hybrid chelator DATA^5m^ ((6-pentanoic acid)-6-(amino)methyl-1,4-diazepinetriacetate))-conjugated kit-type SSTR antagonist ^68^Ga-DATA^5m^-LM4 for PET and evaluated the safety, biodistribution, and preliminary diagnostic efficacy of ^68^Ga-DATA^5m^-LM4 in patients with metastatic NETs.

**Methods:** The DATA^5m^-conjugated form of LM4, was labeled with ^68^Ga. A total of 27 patients (19 men/8 women; mean age 61 years) with histopathologically confirmed well-differentiated NETs underwent ^68^Ga-DATA^5m^-LM4 PET/CT for the staging and restaging or patient selection for PRRT. All the patients underwent PET/CT scans 60 min after intravenous bolus injection of 1.85 MBq (0.05 mCi) per kilogram of body weight (151 ± 54 MBq mean ± SD) of ^68^Ga-DATA^5m^-LM4.

**Results:** DATA5m-LM4 was successfully labeled with ^68^Ga, achieving high yield and purity. After decay correction, radiochemical yields (RCYs) of 80-95% and radiochemical purities (RCP) greater than 98% were obtained. ^68^Ga -DATA^5m^-LM4 was well tolerated in all patients, without clinically relevant adverse effects. A significantly lower uptake in normal liver parenchyma was observed with ^68^Ga-DATA^5m^-LM4 compared to ^68^Ga-DOTA-TATE PET/CT (3.90 ± 0.88 *vs.* 9.12 ± 3.64, P < 0.000001). Additionally, uptake in the thyroid gland, pancreas, and spleen was also lower (P < 0.05). 14 patients underwent ^68^Ga-DOTA-TOC PET/CT. ^68^Ga-DATA^5m^-LM4 uptakes in the liver and spleen were significantly lower than those of ^68^Ga-DOTA-TOC uptake (3.70 ± 0.79 *vs.* 5.33 ± 2.43, P = 0.0397; 11.88 ± 6.88 *vs.* 26.55 ± 16.07, P = 0.0022). Tumor lesions showed high uptake intensity on ^68^Ga-DATA^5m^-LM4 PET/CT, with the highest SUVmax up to 167.93 (mean ± SD, 44.47 ± 36.22). With SUVmean of healthy liver, kidneys, and blood pool as background to normalize the SUVmax of the single most intense lesion, tumor-to-background ratios were 20.32 ± 19.97 (range, 3.40 - 98.78) and 4.30 ± 3.03 (range, 0.65 - 14.70), 38.63 ± 35.97 (range, 4.1 - 173.12), respectively.

**Conclusion:** This study demonstrated that the novel SSTR antagonist ^68^Ga-DATA^5m^-LM4 can be efficiently labeled with high radiochemical yield and purity, supported by a highly convenient production process. The tracer exhibited excellent imaging performance, with a highly favorable biodistribution characterized by high tumor contrast and minimal uptake in normal organs, particularly the liver, enabling superior lesion detection. The practical advantages of this straightforward labeling process, achieved without any apparent loss in diagnostic efficacy, offer a significant benefit over other competing antagonists. The ease of production, including the potential for a “kit-type” labeling method, makes ^68^Ga-DATA^5m^-LM4 an overall extraordinarily promising radiopharmaceutical for the staging and restaging of NET patients.

## Introduction

Neuroendocrine tumors (NETs) are a heterogeneous group of neoplasms arising with predominantly neural and endocrine differentiation that are often able to produce hormones and other biologically active substances 1, 2, arising from any organ where endocrine cells are present and are most commonly found in the gastrointestinal tract, pancreas, and lung 1, 3, 4. To reflect prognosis, NETs are also divided into histologically well- or poorly differentiated 5.

Neuroendocrine tumors are characterized by overexpression of the somatostatin receptor (SSTR), especially SSTR subtype 2 (SSTR2), making them accessible for radiodiagnostic and therapeutic strategies. Currently, Gallium-68 (^68^Ga) labeled SSTR-targeted somatostatin analogs have been used for positron emission tomography-computed tomography (PET/CT) in routine clinical practice for the diagnosis and management of NETs. The U.S. Food and Drug Administration, as well as the European Medicines Agency, have approved ^68^Ga-labeled [^68^Ga]Ga-DOTA-TATE and [^68^Ga]Ga-DOTA-TOC for imaging of SSTR positive neuroendocrine tumors.

DOTA-TOC and DOTA-TATE are comprised of the DOTA-chelator conjugated to 8 cyclized amino acids with high affinity for SSTR2, for which they act as agonists. SSTR agonists readily internalize into tumor cells, allowing tracer accumulation in the target cells. Recent studies have shown that SSTR antagonists are likely to perform better in certain patients, especially with low SSTR2 expression 6, 7. Accordingly, compared to SSTR2 agonist radiotracers complementary SSTR2 antagonist radiotracers such as ^68^Ga labeled, DOTA or NODAGA chelator conjugated to JR11 and LM3 (JR11 = p-Cl-Phe-cyclo(D-Cys-Aph(Hor)-D-Aph(cbm)-Lys-Thr-Cys)D-Tyr-NH2; NODAGA = 1,4,7-triazacyclononane,1-glutaric acid-4,7-acetic acid; LM3 = p-Cl-Phe-cyclo[D-Cys-Tyr-D-4-amino-Phe(carbamoyl)-Lys-Thr-Cys]D-Tyr-NH_2_ have shown higher uptake in preclinical and clinical settings 6, 8-12.

However, there might be options for better-performing SSTR2-antagonists. The chelator and radioisotope greatly influence the affinity and pharmacokinetics of SSTR radiotracers, for example, by lipophilicity and charge of the molecule. The chelator DOTA, for example, is not well suited for complexing the relatively small (radio) metal Gallium and necessitates elevated reaction temperature which is not only detrimental for many antibodies but also small and heat-sensitive biomolecules. Furthermore, often longer reactions are necessary, and additionally, time is required for cooling before intravenous injection, thereby imposing limitations for clinical use due to the short half-life of 67.7 min. DATA (6-amino-1,4-diazepine-triacetic acid) is a novel type of chelator exhibiting cyclic, acyclic, and inter-mediate properties, which have advantageous properties for ^68^Ga-labeling compared with established chelators. In particular, DATA can afford a more rapid quantitative radiolabeling with ^68^Ga at ambient temperature in a less acidic pH range 13. Furthermore, the related chelate AAZTA provides the option for labeling with therapeutic isotopes such as ^177^Lu 14.

In this study, we present another promising candidate for SSTR-antagonist imaging, a novel DATA conjugated SSTR antagonist PET tracer LM4, designated as ^68^Ga-DATA^5m^-LM4. This first-in-human study aimed to establish proof-of-concept and evaluate the feasibility of using this novel SSTR antagonist ^68^Ga-DATA^5m^-LM4 for PET/CT in clinical use. We assessed the production, safety, biodistribution and preliminary diagnostic efficacy of ^68^Ga-DATA^5m^-LM4 in patients with metastatic NETs.

## Materials and Methods

### Patients

From September 2020 to January 2022, twenty-seven patients (19 men /8 women; mean age, 61.0 ± 12.1 years; age range, 38-82 y) with histopathologically confirmed well-differentiated metastatic NETs, who underwent ^68^Ga-DATA^5m^-LM4 PET/CT for disease staging and restaging, were included in this study. All procedures involving human participants were conducted in compliance with the German Medicinal Products Act (section 13, subsection 2b), the 1964 Declaration of Helsinki, and the responsible regulatory body. The study was performed in accordance with German regulations (Federal Agency for Radiation Protection) concerning radiation safety and was approved by the responsible ethical committee for data collection and analysis (Curanosticum Wiesbaden-Frankfurt, Germany). All patients signed a detailed informed consent form and consented to the use of their anonymized clinical data for scientific purposes. To avoid the influence of cold somatostatin analog treatment on imaging, PET scans were scheduled 3-4 weeks after the last dose of long-acting somatostatin analogs. The baseline demographics of the patients are shown in **Tables 1 and 2**.

### Synthesis and radiolabeling

**Figure 1:**
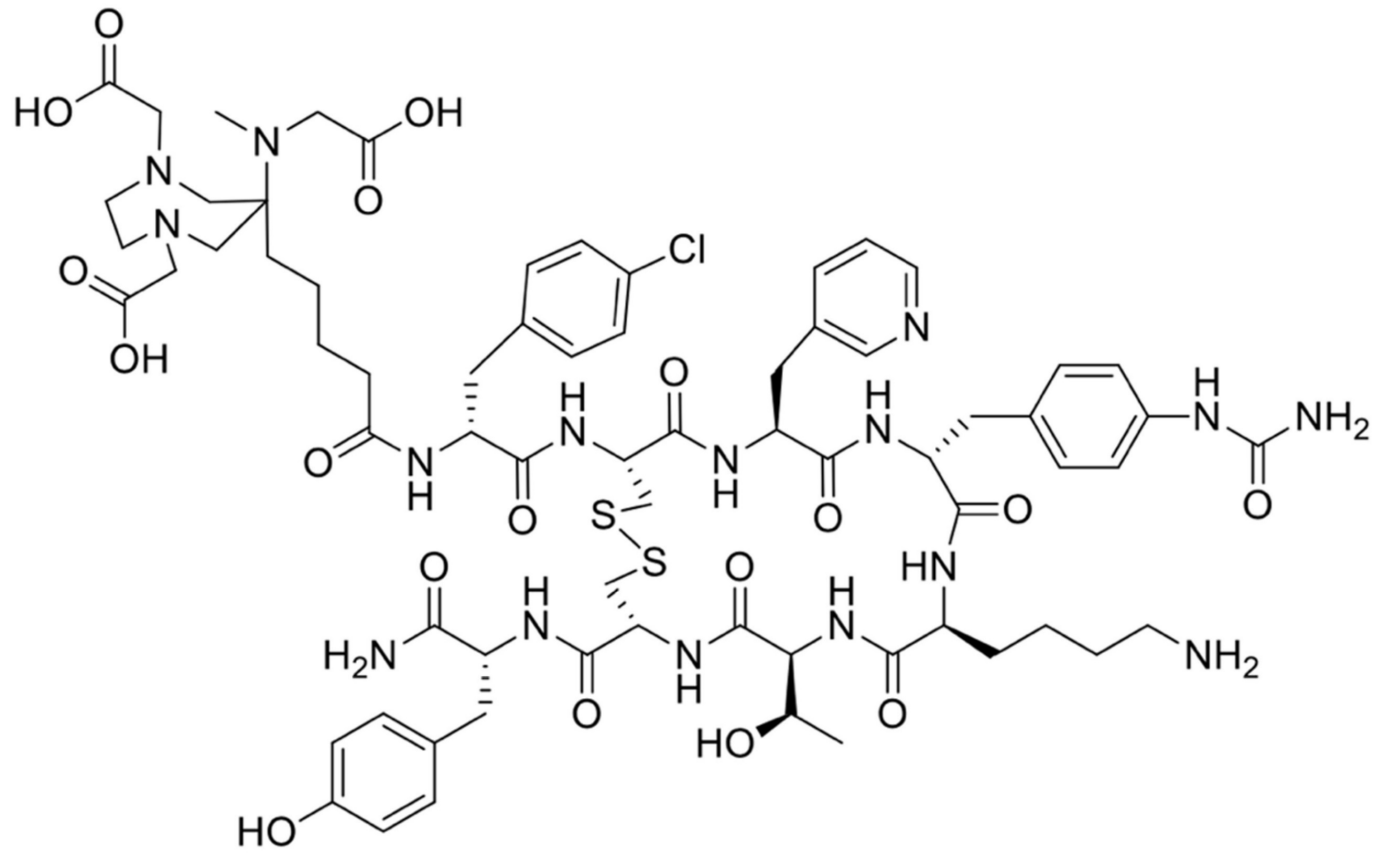
Structure of DATA^5m^-LM4.

The precursor DATA^5m^-LM4, designated for labeling of ^68^Ga and targeting SSTR2, consists of the chelate ((6-pentanoic acid)-6-(amino)methyl-1,4-diazepinetriacetate)) and is conjugated to the SSTR2 ligand LM4 = p-Cl-Phe-cyclo[DCys-Pal-Daph(Cbm)-Lys-Thr-Cys]DTyr-NH_2_ with Pal = Pyridylalanine using a peptide bond (**Figure 1**). Synthesis was performed as previously described 15, 16.

^68^Ga was eluted from a ^68^Ge/^68^Ga generator (Eckhardt und Ziegler) using 0.1M HCl and mixed with sodium acetate buffer to adjust the pH value. 50 µg of the precursor DATA^5m^‑LM4 was added to 1000 µL sodium acetate buffer (pH 5.0), which was then added to the generator eluate. Quality control was performed using analytical high-performance liquid chromatography and thin-layer chromatography.

### PET/CT imaging and evaluation

All the patients underwent PET/CT scans 60 min after intravenous bolus injection of 1.85 MBq (0.05 mCi) per kilogram of body weight (151 ± 54 MBq mean ± SD) of ^68^Ga-DATA^5m^-LM4. Patients received between 0.08 and 0.20 µg/MBq of the peptide which leads to values between 0.14 µg/kg and 0.44 µg/kg. One hour after intravenous administration, PET/CT images were acquired from the vertex to the proximal femora on a Biograph Vision 600 Edge (Siemens Healthineers). A low-dose CT scan was acquired with an automatic tube voltage selection and current modulation using CARE kV and CARE Dose4D (Siemens Healthcare). PET scanning was performed using continuous bed motion, with an average imaging speed of 0.8 mm/sec in the caudocranial direction, resulting in a scan time of approx. 20 min from the vertex to the proximal thighs. PET image reconstruction was performed using point spread function (TrueX) and time of flight modeling, four iterations with five subsets, no filtering was applied (“all-pass”). The images were transferred to an MMWP workstation (Siemens) for analysis. The vital parameters of the patients were measured, and routine blood tests, liver function, and renal function were examined. Any possible side effects during ^68^Ga-DATA^5m^-LM4 PET/CT scanning and within 1 week after the examination were collected and analyzed.

Patients received another ^68^Ga-SSTR PET/CT with ^68^Ga-DOTA-TOC/TATE and/or ^68^Ga-NODAGA-LM3 within 9 months, with PET/CT imaging protocols the same as those for ^68^Ga-DATA^5m^-LM4. Doses were calculated according to the participant's body weight (1.8-2.2 MBq/kg). All PET images were evaluated by two board-certified and experienced nuclear medicine physicians, without being blinded to the medical history of the patients, on the reconstructed images (Affinity Viewer, Hermes Medical Solutions). Disagreements were resolved via consensus.

Visual analysis was used to determine the general biodistribution and the temporal and inter-subject stability. The volume of interest of normal organs/tissues and concerned lesions were drawn on the serial images. The radioactivity concentration and standardized uptake value (SUV) in the volumes of interest were obtained through the software. The physiologic uptake of ^68^Ga-DATA^5m^-LM4 was evaluated in the brain, pituitary gland, parotid gland, thyroid gland, lungs, blood pool, liver, spleen, pancreas, stomach, small intestine, kidneys, adrenal glands, red marrow (vertebrae), and muscle.

Regions of interest were drawn manually on the site of lesions using a 3-dimensional ellipsoid iso-contour on each image with the assistance of the corresponding CT images. Any reasonably non-physiologic focal accumulations were interpreted as tumor lesions. Lesion uptake was measured using SUVmax. Targeted lesions were chosen in each patient, including the lesion with the highest uptake and the second-highest uptake of the tumors. Tumor-to-background ratio (TBR) was quantified using the SUV_mean_ of healthy liver parenchyma, blood pool, and kidney as background reference tissues.

### Statistical analysis

Statistical analysis was performed with Prism 5.0 software (Graph-Pad). Continuous variables were summarized as mean ± SD. Student's t-test was used to compare the SUVs of normal tissues between ^68^Ga-DATA^5m^-LM4 PET and other ^68^Ga-SSTR PET images. All tests were 2-tailed, with the statistical significance defined as* P* < 0.05.

## Results

### Chemistry and radiochemistry

The structure of DATA^5m^-LM4 is presented in **Figure 1.** Radiochemical yields (RCY) of 80-95% were achieved within 15 min at 50 °C, after decay correction. The entire labeling process of ^68^Ga-DATA^5m^-LM4 took approximately 20 min, including heating, purification, filtration, and formulation. Subsequent purification, formulation, and sterile filtration were carried out. Quality control was performed using analytical high-performance liquid chromatography and thin-layer chromatography, both of which demonstrated radiochemical purities (RCP) exceeding 98%. The specific activity was between 12 and 17 MBq/nmol.

### Safety and biodistribution in normal organs

^68^Ga-DATA^5m^-LM4 was well tolerated in all patients, with no clinically significant adverse effects. All observed vital signs (including blood pressure, temperature, and heart rate) were normal at the 4-h follow-up.

**Figure 2:**
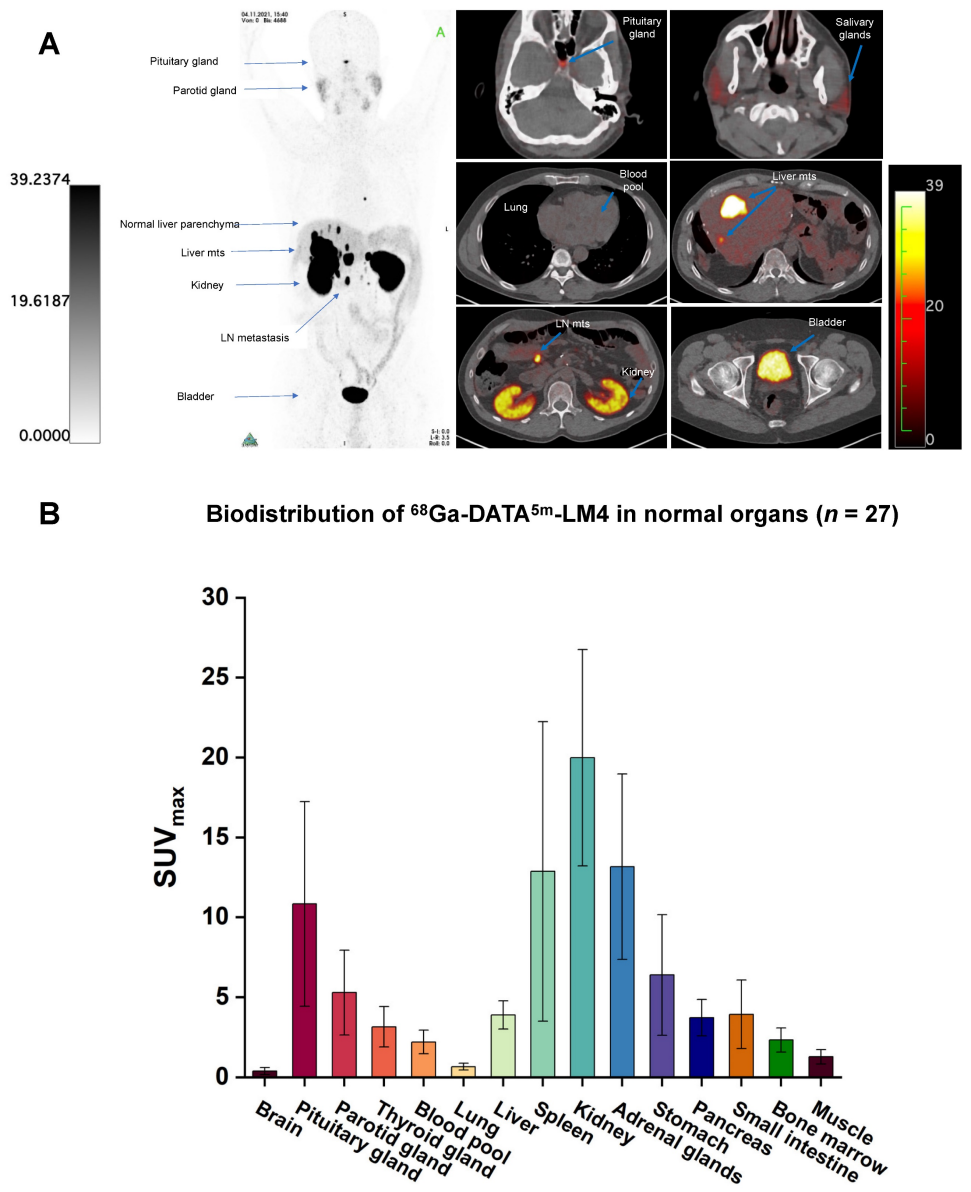
(A) Representative maximum-intensity-projection images fused axial PET/CT images of a 48-y-old patient with well-differentiated, non-functioning neuroendocrine neoplasm of the pancreas with extensive hepatic and lymph node metastases at 60 min after intravenous injection of ^68^Ga-DATA^5m^-LM4. The main regions with prominent ^68^Ga-DATA^5m^-LM4 uptake are the pituitary gland, kidneys, and bladder, as well as the liver metastases and lymph nodes metastases (arrows). There is low background activity in the normal liver parenchyma. (B) PET-based biodistribution analysis of the 27 patients imaged at 1 h after injection.

The biodistribution of ^68^Ga-DATA^5m^-LM4 is shown in **Figure 2**. ^68^Ga-DATA^5m^-LM4 was cleared efficiently after 1 h from the circulation and was excreted mainly through the kidneys and urinary tract. The highest normal organ uptake of ^68^Ga-DATA^5m^-LM4 (except for the kidneys and urinary bladder) was noted in the adrenal glands, followed by the spleen and pituitary gland, with SUV_max_ of 13.16 ± 5.80, 12.87 ± 9.37, and 10.84 ± 6.40 at 60 min after injection, respectively. The parotid gland, thyroid gland, liver, and pancreas showed moderate uptake, with SUV_max_ of 5.29 ± 2.64, 3.16 ± 1.26, 3.90 ± 0.88, and 3.73 ± 1.13, respectively. Uptake in the skeletal system, brain, lungs, blood pool, mediastinum, red marrow, and muscle were at background level.

### Comparative biodistribution between ^68^Ga-DATA^5m^-LM4 and other^ 68^Ga-labeled somatostatin analogs

The comparison of the *in vivo* distribution pattern between ^68^Ga-DATA^5m^-LM4 and that of ^68^Ga labeled other SSTR analogs, including ^68^Ga-DOTA-TATE, ^68^Ga-DOTA-TOC, ^68^Ga-NODAGA-JR11, ^68^Ga-NODAGA-LM3 are shown in **Table 3**. A significantly lower uptake in normal liver parenchyma was observed with ^68^Ga-DATA5m-LM4 compared to ^68^Ga-DOTA-TATE PET/CT (3.90 ± 0.88 *vs.* 9.12 ± 3.64, *P* < 0.000001). Additionally, uptake in the thyroid gland, pancreas, and spleen was also lower (*P* < 0.05).

**Figure 3:**
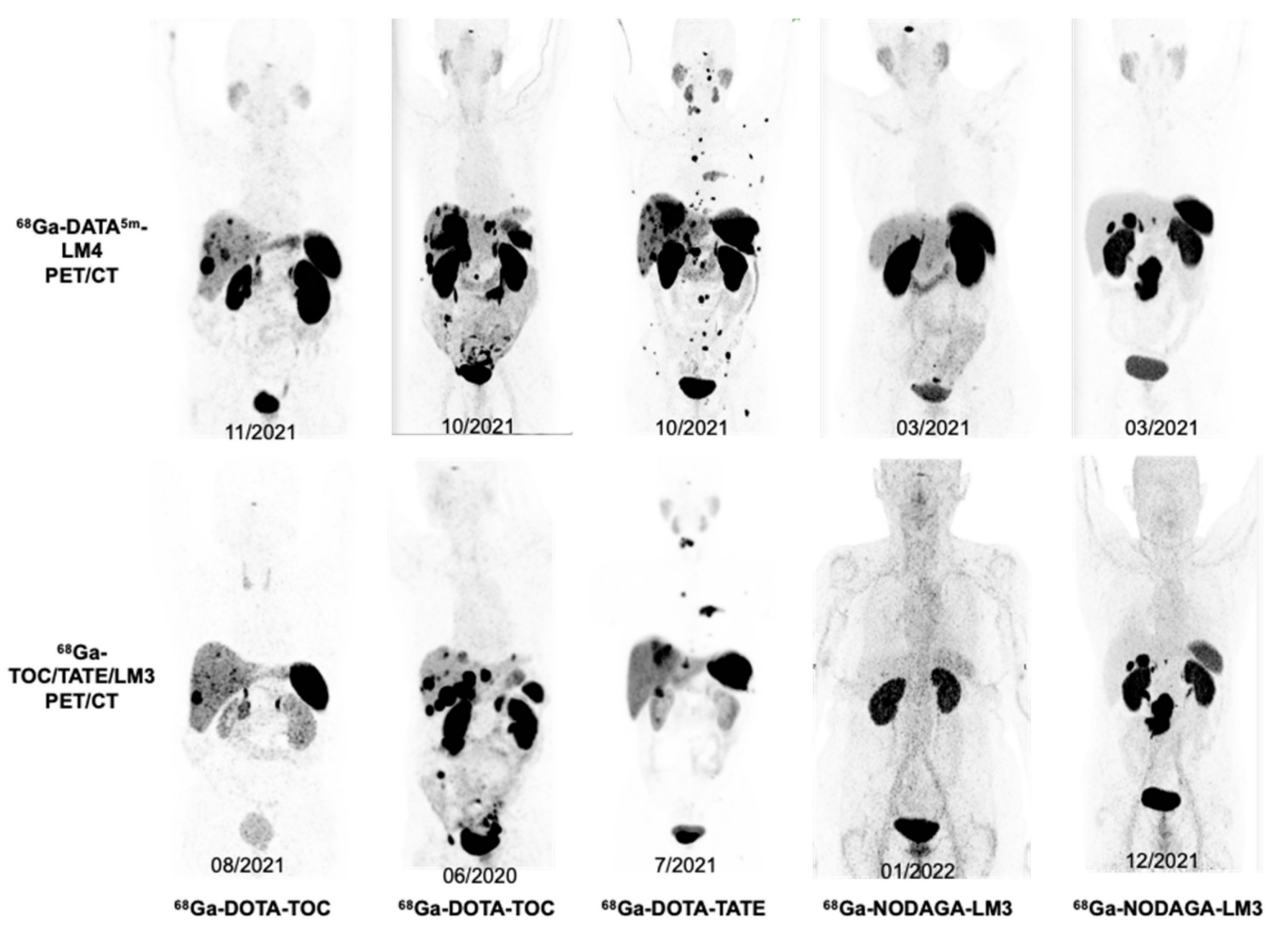
Illustration of image quality of whole-body maximum-intensity projections in 5 representative NET patients (Patients 3, 8, 21, 17, 10) who underwent both ^68^Ga-DATA^5m^-LM4 PET/CT (upper) and other SSTR-targeting ^68^Ga-TOC/TATE/LM3 PET/CT (lower). Patients 3 and 17 underwent 2 cycles PRRT between scans, Patient 21 received treatment with Everolimus, Patients 8 and 10 did not receive any treatments between scans.

**Figure 4:**
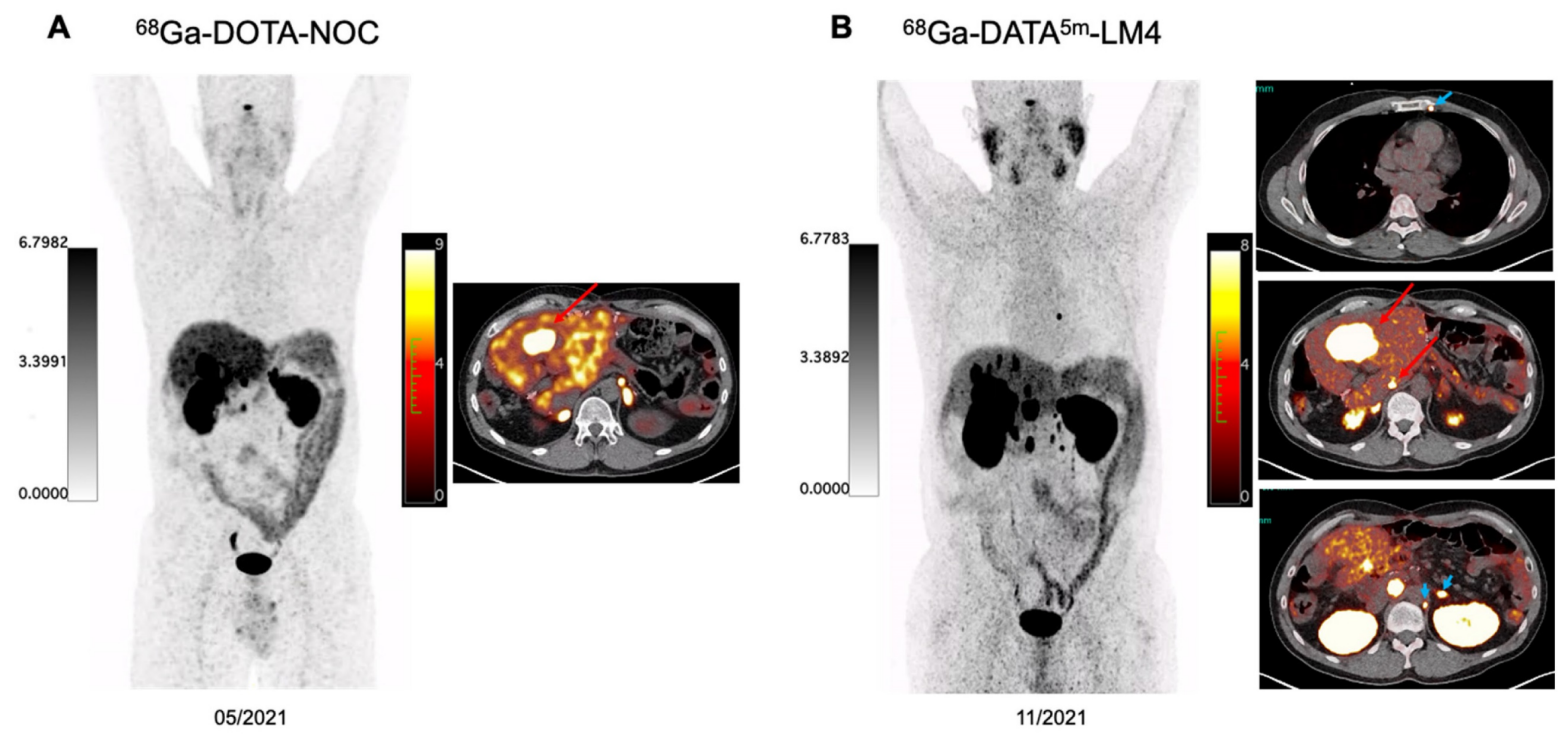
A 48-year-old patient with well-differentiated non-functioning serotonin-producing NET of the pancreas with extensive hepatic and lymph node metastases, G2 with Ki-67 index of 10%, immunohistochemical staining strongly positive for Chromogranin A and Synaptophysin. The patient has previously received treatment with duodenopancreatectomy, splenectomy, segmental liver resection and several hepatic metastectomies, lanreotide, right hemihepatectomy, CAPTEM chemotherapy, and 5 cycles of PRRT since 2017 using ^90^Y- and ^177^Lu-DOTA-TOC, ^177^Lu-DOTA-LM3 by May 2021. The patient received chemotherapy between May 2021 and November 2021. The intraindividual comparison between ^68^Ga-DOTA-NOC and ^68^Ga-DATA^5m^-LM4 PET/CT demonstrated significantly lower uptake in normal liver parenchyma on ^68^Ga-DATA^5m^-LM4 PET/CT resulting in a higher TBR of 10.3 allowing even the detection of very small hepatic metastases (>10 tumor lesions) as compared to ^68^Ga-DOTA-NOC PET/CT (green arrows). Additionally, a tiny left internal mammary lymph node metastasis was clearly visualized on ^68^Ga-DATA^5m^-LM4 PET/CT as well as para-aortic lymph node lesions left to the adrenal gland (blue arrowheads).

Among the 27 patients, 14 also underwent ^68^Ga-DOTA-TOC PET/CT, 1 underwent ^68^Ga-DOTA-TATE PET/CT, and 4 underwent ^68^Ga-NODAGA-LM3 PET/CT (**Table 4** and **Table S1**). The semiquantitative analysis demonstrated that ^68^Ga-DATA^5m^-LM4 uptake in the liver and spleen was significantly lower than the ^68^Ga-DOTA-TOC uptake (3.70 ± 0.79 *vs.* 5.33 ± 2.43, P = 0.0397; 11.88 ± 6.88 *vs.* 26.55 ± 16.07, *P* = 0.0022, respectively). In contrast, the uptake of ^68^Ga-DATA^5m^-LM4 in the kidneys was higher than that of ^68^Ga-DOTA-TOC (19.40 ± 7.12 *vs.* 16.44 ± 6.04, *P* = 0.0059). ^68^Ga-DATA^5m^-LM4 uptake in the pituitary gland was significantly higher than the ^68^Ga-NODAGA-LM3 uptake (8.87 *vs.* 4.79, *P* = 0.0183). Representative images are shown in **Figures 3 and 4**.

### Tumor uptake

**Figure 5:**
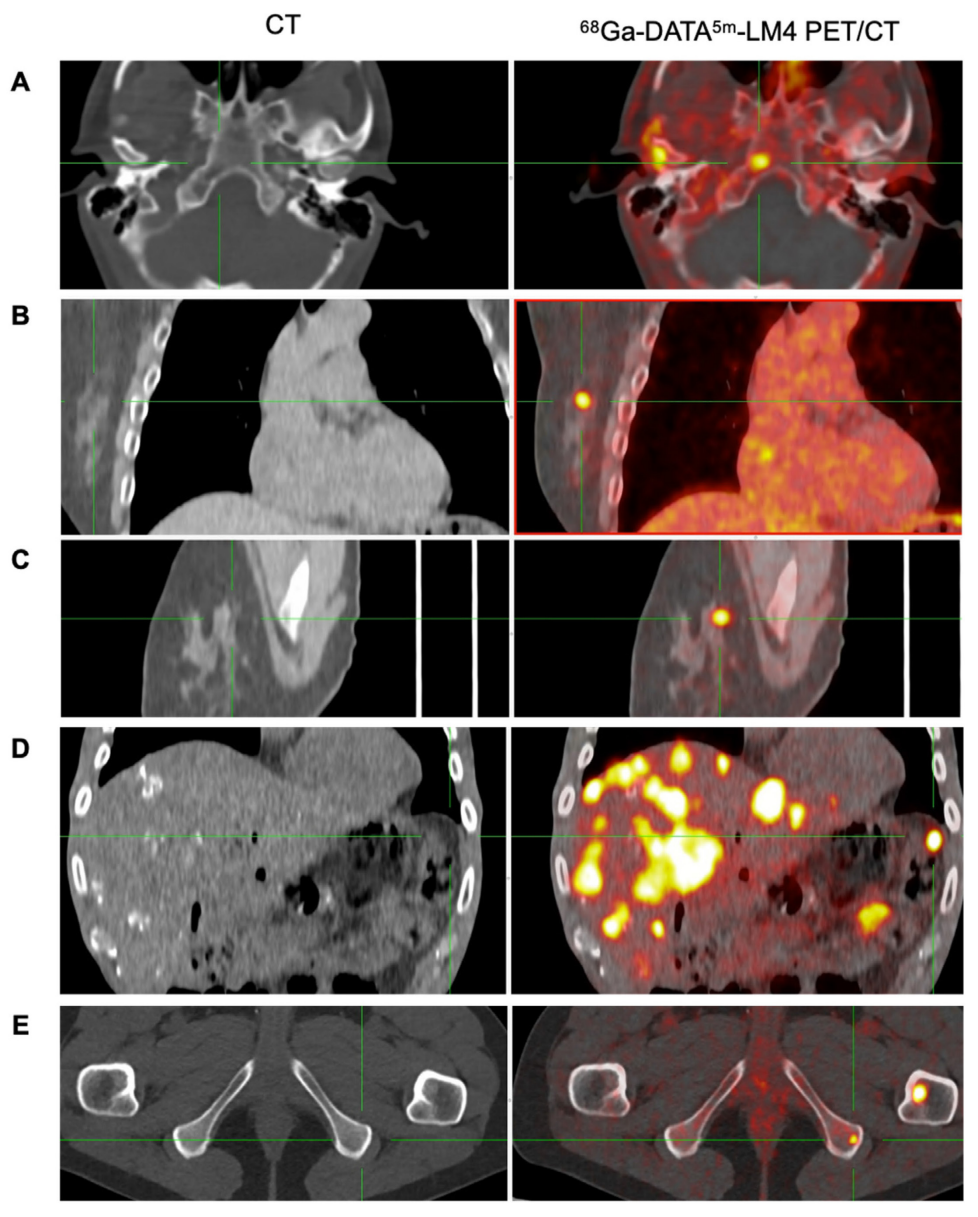
Representative images of ^68^Ga-DATA^5m^-LM4 PET/CT demonstrating the detection of tumor metastases that are undetectable on conventional CT, located in: skull base (A), right breast (B and C), peritoneum (D), and ischial bone (E).

**Figure 6:**
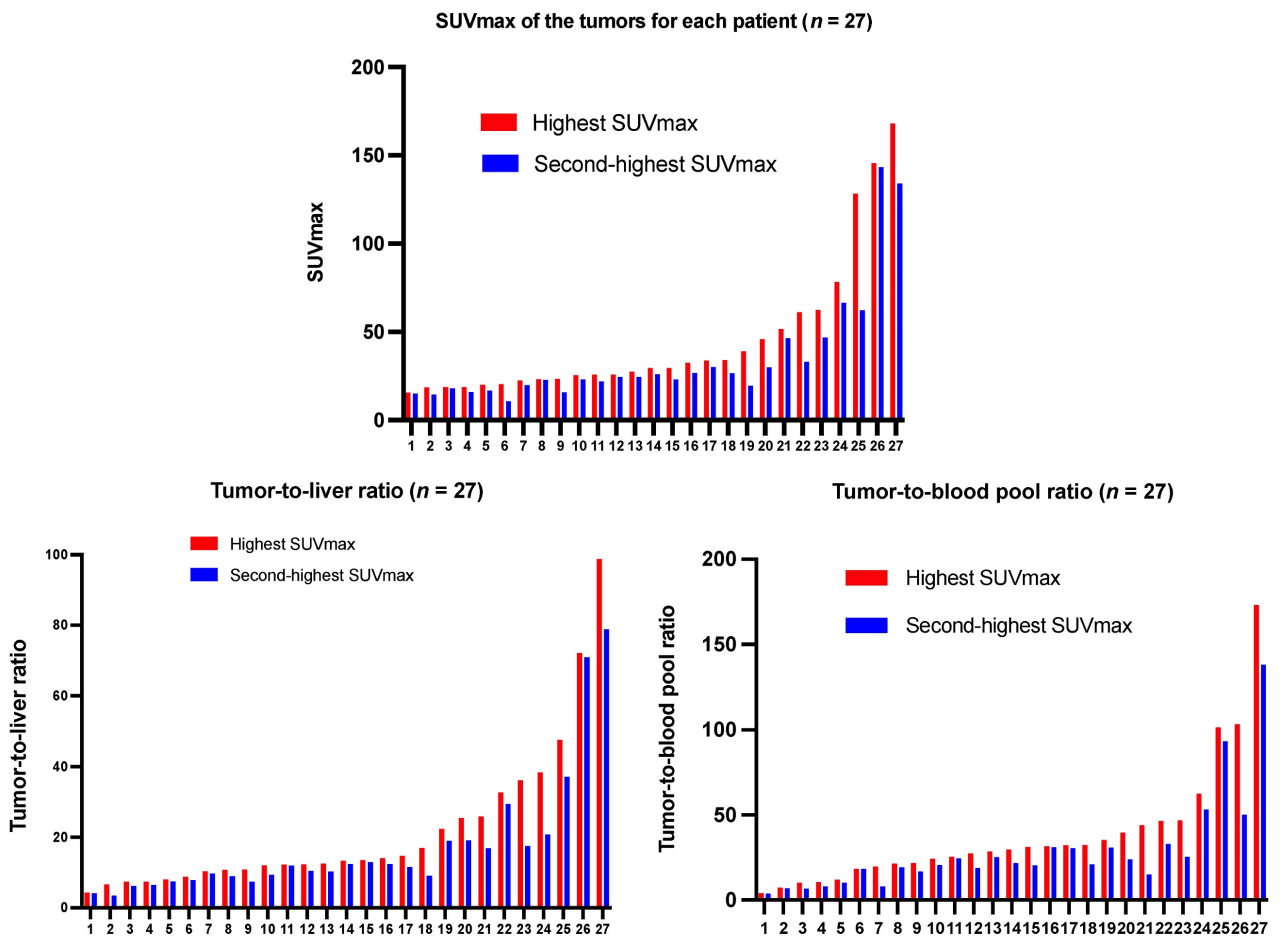
List of SUVmax of tumors, tumor-to-liver ratio, and tumor-to-blood pool ratio in the 27 NET patients in ascending order. Red Bar: Highest SUVmax and TBR according to highest tumor uptake detected on ^68^Ga-DATA^5m^-LM4 PET/CT; Blue bar: The second-highest SUVmax and TBR in each patient.

^68^Ga-DATA^5m^-LM4 PET/CT effectively detected tumor lesions with high uptake, including small lesions not visible on CT. Representative images are shown in **Figure 5**. The highest uptake and the second highest uptake of the tumor lesions in each patient ranged from 12.30 - 167.93 (mean ± SD, 44.47 ± 36.22) and 10.4 - 133.99 (mean ± SD, 34.00 ± 27.43), respectively (**Figure 6**).

Liver metastases, lymph node metastases, bone metastases, and other metastases were detected in 22 (81.5%), 16 (59.3%), 13 (48.1%), and 16 (59.3%) patients. The SUV_max_ of lesions in the liver, lymph nodes, bone, and other sites (pancreas, lung, peritoneum, spleen, subcutaneous pelvis, breast, skull base or glomus tympanicum, duodenum, stomach, cardia, intestines, abdominal wall, glomus caroticum, paratracheal, mesenterium, infradiaphragmal area, soft tissue dorsal to uterus, peritoneum or ovary) was 36.17 ± 30.39 (range, 4.30 - 128.30), 29.19 ± 18.95 (range, 3.80 - 62.30), 32.83 ± 45.81 (range, 2.2 - 167.93), 22.92 ± 21.79 (range, 4.30 - 93.4), respectively. Tumor-to-background ratios relative to the liver and blood pool were 20.32 ± 19.97 (range, 3.40 - 98.78) and 38.63 ± 35.97 (range, 4.1 - 173.12), respectively.

## Discussion

In this study, we conducted the first clinical investigation using the novel SSTR antagonist ^68^Ga-DATA^5m^-LM4 in patients with metastatic NETs. We demonstrated that ^68^Ga-DATA^5m^-LM4 could be synthesized with high yield and fast radiolabeling with ^68^Ga. ^68^Ga-DATA^5m^-LM4 was safe and well-tolerated in all subjects. In patients with disseminated metastases NETs, it showed a superior biodistribution with very high tumor contrast and low uptake in the spleen, pancreas, and especially in the liver as compared to the agonistic SSTR ligands, such as ^68^Ga-DOTA-TATE, which facilitates favorable lesion detection with ^68^Ga-DATA^5m^-LM4.

Several preclinical studies have shown that radiolabeled SSTR antagonists exhibit higher uptake in SSTR-expressing tumors compared to SSTR agonists 6, 17. The first clinical evaluation of SSTR antagonists confirmed these findings, demonstrating higher tumor uptake and better tumor-to-background ratios with the antagonist ^111^In-DOTA-BASS compared to the SSTR agonist ^111^In-DTPA-octreotide 8. Clinically, SSTR antagonists have demonstrated higher sensitivity and better image contrast. ^68^Ga-NODAGA-JR11 has in regard to this exhibited superiority compared with the agonist ^68^Ga-DOTA-TOC in patients with GEP-NETs 9, 18, and similarly, the diagnostic efficacy superiority of the antagonist ^68^Ga-NODAGA-LM3 was shown when compared to agonist ^68^Ga-DOTA-TATE in NETs 11. More recently, ^68^Ga-NODAGA-JR11 (also known as ^68^Ga-SSO-120) has shown high diagnostic value in small cell lung cancer (SCLC) 19, 20, a tumor type with lower SSTR expression than NETs, highlighting its potential for theranostics applications.

The chelator DOTA can severely affect the affinity and pharmacokinetics of the SSTR ligand, which is not well suited for complexing the relatively small (radio) metal ^68^Ga and also overcompensates the charge of the metal leaving the metal with an overall charge of minus one. For SSTR antagonists, Fani *et al.* have demonstrated in preclinical experiments that the affinity and pharmacokinetics of the radiolabeled somatostatin-based antagonists strongly depend on the chelator and radiometal and reported ^68^Ga-NODAGA-LM3 has a 10-fold higher SSTR2 affinity than ^68^Ga-DOTA-LM3 21. Zhu *et al.* reported in a head-to-head comparison that ^68^Ga-DOTA-JR11 was superior to ^68^Ga-DOTA-TATE in the detection of liver metastases but much less sensitive for bone metastases, and probably limited its role as a diagnostic pair for the theranostic approach due to the lower somatostatin receptor subtype 2 affinity 22. These findings emphasize the importance of developing novel chelates to further improve image contrast, and the search for novel tracers with even higher affinity and practicality is thus forthgoing and forthcoming.

DATA is a novel type of chelator exhibiting cyclic, acyclic, and intermediate properties, which have advantageous properties for ^68^Ga-labeling compared to established chelators. ^68^Ga-DATA chelates are immune against trans-chelation (DTPA and apo-transferrin) and trans-metalation (FeIII) and show high stability against human serum and different buffers 23. Furthermore, in contrast to ^68^Ga-DOTA radiotracers, labeling of DATA can be realized at ambient temperature, and therefore, labeling of heat-sensitive molecules is possible. Additionally, no time for cooling before the intravenous injection is needed, thereby overcoming the limitations for clinical use due to the short half-life of ^68^Ga. DATA can afford rapid quantitative radiolabeling with ^68^Ga at ambient temperature in a wide pH range, which greatly promotes convenience for clinical use. Furthermore, it only has three attached carboxylic acids to neutralize the charge of Ga(III), adding no additional charge to the molecule like DOTA and therefore acts similarly to the NODAGA derivatives.

The biodistribution of ^68^Ga-DATA^5m^-LM4 was similar to that of SSTR agonists and antagonists in SSTR_2_-rich organs 9, 11, 22, 24-26. As compared to SSTR agonists ^68^Ga-DOTA-TATE and ^68^Ga-DOTA-TOC, the background uptake of ^68^Ga-DATA^5m^-LM4 in the liver was significantly lower, resulting in high tumor contrast for the liver metastases and significantly higher tumor-to-liver ratios. Lower organ uptake was also observed in the spleen of ^68^Ga-DATA^5m^-LM4 as compared to both ^68^Ga-DOTA-TATE in the literature and ^68^Ga-DOTA-TOC in the head-to-head comparison of the present study. The bone marrow uptake and kidney uptake of ^68^Ga-DATA^5m^-LM4 were higher than those of the agonist ^68^Ga-DOTA-TATE. This suggests the need for dosimetry studies, as bone marrow and kidneys are critical dose-limiting organs in subsequent PRRT, particularly given that bone marrow toxicity is a limiting factor in the application of SSTR antagonist radionuclide therapy. Among SSTR antagonists, studies have indicated that ^68^Ga-NODAGA chelates, for example, ^68^Ga-NODAGA-LM3, achieve higher tumor uptake and greater uptake in SSTR2-positive organs compared to ^68^Ga-DOTA-LM3 27. Therefore, we directly compared ^68^Ga-DATA^5m^-LM4 with a ^68^Ga-NODAGA chelated SSTR antagonist. As compared to other SSTR antagonists like ^68^Ga-NODAGA-JR11 and ^68^Ga-NODAGA-LM3, ^68^Ga-DATA^5m^-LM4 demonstrated a similar biodistribution but showed higher uptake in representative SSTR2-rich organs such as the pituitary and adrenal glands. The reported liver biodistribution for SSTR antagonists has shown variability in the literature 7, 9, 11, 19, 28-30. In our study, ^68^Ga-DATA^5m^-LM4 demonstrated lower liver uptake than previously reported but showed a higher background uptake in the liver, spleen, and kidneys in a direct head-to-head comparison with ^68^Ga-NODAGA-LM3 PET/CT conducted at our center, involving four patients. Future prospective studies with larger cohorts of NET patients are warranted to validate these findings and facilitate a more rigorous head-to-head comparison of these promising radiopharmaceuticals.

Our previous study on ^177^Lu chelated by an antagonist, ^177^Lu-DOTA-LM3, as a treatment showed promising results. It was well-tolerated by the majority of patients, even at a higher radiation dose than that used with SSTR agonists 31. Moreover, it was effective in treating metastatic NETs in patients with low or no tumor uptake of an SSTR agonist. In the present study, ^68^Ga-DATA^5m^-LM4 showed high tumor uptake, with the highest SUV_max_ up to 167.93 (44.47 ± 36.22) and the second highest SUV_max_ up to 133.99 (34.00 ± 27.43), comparable to those for SSTR agonists and other SSTR antagonists 8, 9, 11, 32-34. The high tumor uptake also provides the basis for the application of comparable ligands coupled to, e.g., AAZTA (1,4-bis (carboxymethyl)-6-[bis (carboxymethyl)]amino-6-methylperhydro-1,4-diazepine) to be used as a therapeutic agent for tumor-targeted peptide receptor radionuclide therapy of NETs after being labeled with β- emitters. Preliminary observations indicated that ^68^Ga-DATA^5m^-LM4 PET/CT efficiently detected tumor lesions, including very small metastases and CT-invisible tumors. The practical advantages of this straightforward labeling process, achieved without any apparent loss in diagnostic efficacy, distinguish ^68^Ga-DATA^5m^-LM4 from competing antagonists. The capability to label DATA at ambient temperature in a "kit-type" manner offers a significant advantage over other competing antagonists like DOTA- or NODAGA- labeled JR11/LM3. The advantages, along with the very convenient production and the option for a “kit-type” labeling, make ^68^Ga-DATA^5m^-LM4 an overall extraordinarily promising radiopharmaceutical for the staging and restaging of NET patients.

Compared to macrocyclic chelators based on the cyclen scaffold (e.g., DOTA), DATA chelators facilitate quantitative radiolabeling more rapidly and under milder conditions. Several studies have demonstrated the excellent labeling properties of DATA-conjugated derivatives. For instance, each of the DATA^X^ chelators shows remarkable radiolabeling characteristics, achieving > 95% RCYs over a pH range of 4-7 within 3 minutes using just 15 nmol (10.7 mm) of the chelator at room temperature. In contrast, the current industry standard, DOTA (5-10 mm), requires 5-15 minutes at 80-95 °C to achieve similar RCYs. This combination of superior reaction rate and room-temperature labeling is particularly attractive for a kit-type approach to radiolabeling. The ability to label over a wide pH range is advantageous, as it offers a more robust labeling reaction at a pH suitable for *in vivo* administration, thus saving time, and may also enable the synthesis of previously inaccessible pH-sensitive biomolecules 13. Additionally, the benefit of this molecule is that, in general, it allows for labeling without heat in a single vial with a high formulation pH - an advantage over the SOMAKIT, although we did not demonstrate this in the present first-in-human study due to regulatory constraints.

This study suffers from a few limitations. The patient number is relatively small for definite assessment of this novel radiotracer, and only a few patients underwent paired ^68^Ga-DATA^5m^-LM4 and ^68^Ga-DOTA-TOC PET/CT imaging or paired ^68^Ga-DATA^5m^-LM4 and ^68^Ga-NODAGA-LM3 PET/CT imaging. For the patients who underwent paired PET/CT imaging for biodistribution, the results of the statistical analysis are questionable, due to the small number of patients investigated and the potential influence of the tumor uptakes. Another limitation is the lack of histologic confirmation of the detected metastases or other imaging modalities, although ^68^Ga-SSTR PET has been clinically established as superior for NET diagnosis. Furthermore, potential disease progression or remission between scans, particularly with longer intervals, could influence the results, thereby limiting the ability to draw definitive conclusions about the sensitivity or superiority of lesion detection. Prospective studies with larger patient populations and head-to-head comparisons are warranted in the future to better explore the role of ^68^Ga-DATA^5m^-LM4 and the potential superiority of ^68^Ga-DATA^5m^-LM4 in tumor diagnosis for NET patients.

## Conclusion

This study indicated that the novel SSTR antagonist ^68^Ga-DATA^5m^-LM4 can be efficiently labeled with high radiochemical yield and purity, supported by a highly convenient production process. The tracer exhibited excellent imaging performance, with a highly favorable biodistribution characterized by very high tumor contrast and minimal uptake in normal organs, particularly the liver, enabling superior lesion detection. The practical advantages of this straightforward labeling process, achieved without any apparent loss in diagnostic efficacy, distinguish ^68^Ga-DATA^5m^-LM4 from other competing antagonists. Prospective studies with a head-to-head comparison between ^68^Ga-DATA^5m^-LM4 and other compounds are warranted to better explore the role of ^68^Ga-DATA^5m^-LM4 for NET patients.

## Supplementary Material

Supplementary table.

## Figures and Tables

**Table 1:** Demographic and Characteristics of Patients with NETs (*n* = 27)

Characteristic	Number (*n*)	Percentage (%)
Age at scanning (y)		
Mean ± SD	61.0 ± 12.1	
Range	38 - 82	
Sex		
Male	19	
Female	8	
Primary tumor site		
Pancreas	12	44.4
Ileum	9	33.3
Duodenum	1	3.7
Midgut	1	3.7
Others	4	14.8
Ki-67 grading		
G1	6	22.2
G2	15	55.6
G3	3	11.1
Ki-67 not available	3	11.1
^68^Ga-DATA^5m^-LM4 PET imaging		
Liver mts	22	81.5
LN mts	15	55.6
Bone mts	11	40.7
Other mts	13	48.1

**Table 2:** Patient Characteristics

No.	Sex	Age	Primary tumor	Differentiation	Functional	Grade	Ki-67	Liver mts	LN mts	Bone mts	Other mts	Chromogranin A	Synaptophysin
1	M	39	Pancreas	Well	No	G 2	10%	yes	yes	yes	Peritoneal	/	/
2	F	38	Kidney	Well	No	/	/	yes	yes	no		/	/
3	M	69	Ileum	Well	Yes	G 1	<1 %	yes	yes	no		positive	positive
4	F	78	Thyroid	/	/	/	/	no	yes	yes		/	/
5	F	76	Ileum	Well	Yes	G 1	2%	yes	no	no	Peritoneal	positive	positive
6	F	71	Lung	Well	No	G 2	20%	yes	no	yes		positive	positive
7	M	59	Pancreas	Well	No	G 3	47%	yes	no	yes		positive	positive
8	M	74	Ileum	Well	Yes	G 1	1%	yes	yes	no	Peritoneal	/	positive
9	M	65	Midgut	Well	Yes	G 1	<1%	yes	yes	no	Pleural	/	/
10	M	54	Ileum	Well	No	G 2	5%	yes	yes	no		positive	/
11	M	82	Pancreas	Well	No	G 2	4%	yes	yes	yes	Peritoneal	positive	positive
12	M	60	Paraganglioma	/	No	G 2	10-20 %	no	no	yes	Adrenal glands	positive	positive
13	M	60	Pancreas	Well	Yes	G 2	10%	yes	yes	no		/	/
14	M	52	Pancreas	Well	Yes	G 2	5%	no	yes	no	Stomach, spleen, adrenal gland	positive	positive
15	M	49	Pancreas	Well	No	G 2	10%	yes	yes	no		positive	positive
16	M	78	Pancreas	Well	No	/	/	no	yes	no		/	/
17	F	62	Ileum	Well	Yes	G 2	10%	yes	no	no	Ovaries, small intestinal mesentery, peritoneal	positive	positive
18	M	73	Ileum	Well	Yes	G 2	15%	yes	yes	yes	Lung, peritoneal	positive	positive
19	M	53	Ileum	Well	Yes	G 2	15%	yes	no	no	Peritoneal	/	/
20	M	59	Pancreas	Well	Yes	G 3	52%	yes	no	no	Peritoneal	/	/
21	M	38	Duodenum	Well	No	G 3	50%	yes	no	yes		positive	positive
22	M	69	Ileum	Well	Yes	G 2	4%	yes	no	yes	Peritoneal, lateral abdominal wall, small intestinal mesentery, mediastinal LK metastasis	/	/
23	F	60	Ileum	Well	Yes	G 2	12%	no	no	no	Breasts, omentum, intramuscular	/	/
24	F	60	Pancreas	Well	No	G 1	1-2 %	yes	no	no		positive	positive
25	F	52	Pancreas	Well	No	G 1	1-2 %	yes	no	no		/	/
26	M	57	Pancreas	Well	No	G 2	13%	yes	yes	yes		positive	positive
27	M	62	Pancreas	Well	Yes	G 1	<2 %	yes	yes	yes		positive	positive

**Table 3:** Biodistribution of ^68^Ga-DATA^5m^-LM4 in patients with NETs (*n* = 27) and the comparison with other ^68^Ga -SSTR PET

		SUVmax						
	^68^Ga-DATA^5m^-LM4 (*n* = 27)	^68^Ga-DOTA-TATE (*n* = 120) (26)		^68^Ga-DOTA-TATE (*n* = 31) (22)	^68^Ga-DOTA-TOC (*n* = 14) (35)	^68^Ga-NODAGA-JR11 (*n* = 12) (9)	^68^Ga-NODAGA-LM3 (*n* = 8) (11)	^68^Ga-DOTA-NOC (*n* = 89) (25)
Brain	0.39 ± 0.22	<1						
Pituitary gland	10.84 ± 6.4	9.74 ± 3.86	P=0.245004	7.7 ± 3.2		5.8 ± 1.8	9.6 ± 3.5	2.6 ± 1.3
Parotid gland	5.29 ± 2.64	3.17 ± 1.47	P<0.000001			3.7 ± 1.9	2.4 ± 0.9	
Thyroid gland	3.16 ± 1.26	4.18 ± 1.9	P=0.008759		4.2 ± 0.9	2.3 ± 1.2	1.9 ± 0.6	3.4 ± 1.4
Blood pool	2.21 ± 0.73						1.3 ± 0.5	2.6 ± 1.2
Lung	0.66 ± 0.21	0.59 ± 0.28	P=0.223454			1.7 ± 0.6	1.0 ± 0.3	0.9 ± 0.8
Liver	3.90 ± 0.88	9.12 ± 3.64	P<0.000001	9.7 ± 3.0	6.0 ± 1.6	3.2 ± 0.8	6.4 ± 1.8	6.9 ± 2.0
Spleen	12.87 ± 9.37	24.67 ± 8.05	P<0.000001	22.5 ± 8.0	5.1 ±1.3	11.7 ± 4.2	17.5 ± 7.7	22 ± 10
Kidney	19.98 ± 6.77	14.30 ± 4.55	P<0.000001	14.6 ± 3.8	15.8 ± 7.7		17.9 ± 2.7	12.9 ± 3.8
Adrenal glands	13.16 ± 5.80	13.73 ± 5.16	P=0.613082	11.3 ± 4.4	10.1 ± 6.4	8.3 ± 3.9	11.2 ± 4.8	6.0 ± 2.5
Stomach	6.39 ± 3.77			7.1 ± 4.2		4.2 ± 2.3	3.0 ± 0.9	
Pancreas	3.73 ± 1.13	4.69 ± 1.86	P=0.011086	4.3 ± 1.9		3.2 ± 2.0	3.7 ± 1.6	5.8 ± 2.0
Small intestine	3.93 ± 2.14			6.1 ± 1.8		3.5 ± 1.3	3.2 ± 0.7	2.3 ± 1.0
Bone marrow	2.33 ± 0.76	<1		1.6 ± 0.6				0.8 ± 0.3
Muscle	1.28 ± 0.45	<1						

**Table 4:** Uptake of normal organs in patients with a head-to-head comparison between ^68^Ga-DATA^5m^-LM4 PET/CT and ^68^Ga-DOTA-TOC PET/CT (*n* = 14)

	SUVmax		
	^68^Ga-DATA^5m^-LM4	^68^Ga-DOTA-TOC	*P*
Brain	0.41 ± 0.30	0.38 ± 0.20	0.6678
Pituitary gland	8.86 ± 5.29	8.85 ± 4.84	0.9985
Parotid gland	5.19 ± 2.86	2.34 ± 0.97	0.0035
Thyroid gland	2.73 ± 1.03	4.11 ± 3.16	01952
Blood pool (left ventricle)	2.15 ± 0.82	1.65 ± 0.68	0.0038
Lung	0.58 ± 0.22	0.53 ± 0.24	0.6683
Liver	3.70 ± 0.79	5.33 ± 2.43	0.0397
Spleen	11.88 ± 6.88	26.55 ± 16.07	0.0022
Kidney	19.40 ± 7.12	16.44 ± 6.04	0.0059
Adrenal glands	13.13 ± 5.42	9.44 ± 3.14	0.1177
Stomach	6.77 ± 3.16	3.23 ± 1.35	0.0006
Pancreas	3.04 ± 0.74	2.93 ± 0.72	0.3368
Small intestine	4.03 ± 2.20	1.98 ± 0.70	0.0116
Bone marrow (L4-5 vertebra)	2.03 ± 0.63	1.50 ± 0.38	0.0112
Muscle	1.18 ± 0.37	1.27 ± 0.71	0.6563

## Abbreviations

SSTR: somatostatin receptor
NET: neuroendocrine tumors
PET/CT: positron emission tomography combined with computed tomography
GEP-NETs: gastroenteropancreatic neuroendocrine tumors
SSTR2: somatostatin receptor subtype-2
DATA: (6-amino-1,4-diazepine-triacetic acid)
DOTA: 1,4,7,10-tetraazacyclododecane-N,N',N'',N'''-tetraacetic acid
LM4: p-Cl-Phe-cyclo[DCys-Pal-Daph(Cbm)-Lys-Thr-Cys]DTyr-NH2
JR11: p-Cl-Phe-cyclo(D-Cys-Aph(Hor)-D-Aph(cbm)-Lys-Thr-Cys)D-Tyr-NH2
NODAGA: 1,4,7-triazacyclononane,1-glutaric acid-4,7-acetic acid
LM3: p-Cl-Phe-cyclo[D-Cys-Tyr-D-4-amino-Phe(carbamoyl)-Lys-Thr-Cys]D-Tyr-NH2
RCY: radiochemical yields
RCP: radiochemical purities
SUV: standardized uptake value
TBR: tumor-to-background ratio
