# Supplementary material for: First-in-human study of an optimized, potential kit-type, SSTR antagonist 68Ga-DATA5m-LM4 in patients with metastatic neuroendocrine tumors
Source: Theranostics. 2025 Jan 20;15(6):2510–22. doi: 10.7150/thno.94521 (PMC11840726; doi:10.7150/thno.94521)
Supplement: Supplementary file 1 — Supplementary table. [file thnov15p2510s1.pdf]

First-in-human study of an optimized, potential kit-type, SSTR antagonist ^{68}Ga-DATA^{5m}-LM4 in patients with metastatic neuroendocrine tumors

TABLE S1 Uptake of normal organs in patients with a head-to-head comparison between ^{68}Ga-DATA^{5m}-LM4 PET/CT and ^{68}Ga-NODAGA-LM3 PET/CT (n = 4)

	SUVmax		
	**^{68}Ga-DATA^{5m}-LM4**	**^{68}Ga-NODAGA-LM3**	***P***
Brain	0.29 ± 0.09	0.59 ± 0.35	0.1389
Pituitary gland	8.87 ± 2.18	4.79 ± 1.54	0.0183
Parotid gland	5.01 ± 1.96	3.30 ± 0.86	0.0834
Thyroid gland	2.82 ± 0.53	3.82 ± 1.15	0.1168
Blood pool (left ventricle)	1.89 ± 0.20	2.42 ± 0.66	0.2968
Lung	0.55 ± 0.08	0.98 ± 0.26	0.0382
Liver	3.58 ± 0.84	2.46 ± 0.82	0.0415
Spleen	14.59 ± 6.14	5.71 ± 2.71	0.2685
Kidney	18.45 ± 6.50	10.84 ± 0.97	0.0799
Adrenal glands	12.97 ± 5.56	7.42 ± 2.41	0.7162
Stomach	5.28 ± 2.38	3.13 ± 1.08	0.1676
Pancreas	3.67 ± 0.73	2.48 ± 0.55	0.4152
Small intestine	3.36 ± 0.55	2.72 ± 0.64	0.4044
Bone marrow (L4-5 vertebra)	2.01 ± 0.39	1.84 ± 0.59	0.6307
Muscle	0.93 ±0.12	1.06 ± 0.28	0.2883
